# Molecular characterization of long-term survivors of hepatocellular carcinoma

**DOI:** 10.18632/aging.202615

**Published:** 2021-03-03

**Authors:** Junwei Shen, Jing Hu, Jiawen Wu, Xiaoli Luo, Yanfei Li, Jue Li

**Affiliations:** 1 Key Laboratory of Arrhythmias of the Ministry of Education of China, Shanghai East Hospital, Tongji University School of Medicine, Shanghai 200120, China; 2 Shanghai Pudong New Area Mental Health Center, Tongji University School of Medicine, Shanghai 200124, China; 3 Department of Gynecology, Shanghai First Maternity and Infant Hospital, Tongji University School of Medicine, Shanghai 201204, China; 4 Shanghai Key Laboratory of Molecular Imaging, Shanghai University of Medicine and Health Sciences, Shanghai 201318, China

**Keywords:** hepatocellular carcinoma, prognostic marker, G1/S transition, CDK4, TP53 mutation

## Abstract

Hepatocellular carcinoma is one of the most fatal cancers, and the majority of patients die within three years. However, a small proportion of patients overcome this fatal disease and survive for more than five years. To determine the molecular characteristics of long-term survivors (survival ≥ 5 years), we analyzed the genomic and clinical data of hepatocellular carcinoma patients from The Cancer Genome Atlas and the International Cancer Genome Consortium databases, and identified molecular features that were strongly associated with the patients’ prognosis. Genes involved in the cell cycle were expressed at lower levels in tumor tissues from long-term survivors than those from short-term survivors (survival ≤ 1 years). High levels of positive regulators of the G_1_/S cell cycle transition (cyclin-dependent kinase 2 [*CDK2*], *CDK4*, Cyclin E2 [*CCNE2*], *E2F1*, *E2F2*) were potential markers of poor prognosis. Hepatocellular carcinoma patients with *TP53* mutations were mainly belonged to the short-term survivor group. Abemaciclib, an FDA-approved selective inhibitor of CDK4/6, inhibited the cell proliferation and tumor growth of hepatocellular carcinoma cells *in vitro* and *in vivo*. Thus, high G_1_/S transition-related gene levels and *TP53* mutations are promising diagnostic biomarkers for short-term survivals, and abemaciclib may be a potential targeted drug for hepatocellular carcinoma.

## INTRODUCTION

The incidence of liver cancer has recently been increasing rapidly, by about 3% in women and 4% in men each year [1]. Hepatocellular carcinoma (HCC) is the second leading cause of cancer-related mortality, accounting for the deaths of 21,600 men and 10,180 women annually [1, 2]. Notably, the five-year survival rate of HCC patients is only 18% in the United States [1]. Although most HCC patients die within three years, some patients overcome this fatal disease and survive for more than 5 years. Understanding the mechanisms that promote long-term survival (LTSs, survival ≥ 5 years) would be very valuable for the diagnosis and treatment of HCC [3]; however, little is known about the molecular characteristics of LTSs.

Numerous previous studies have identified genes associated with HCC oncogenesis, including *NOTCH2* and *β-CATENIN* [4–6]; however, most of these studies have focused on differentially expressed genes (DEGs) between normal and tumor tissues. For example, *NOTCH2* was reported to be highly expressed in HCC tumor tissues [4], and Yes-associated protein (a member of the Hippo signaling pathway) was found to induce the oncogenesis and excessive growth of HCC [7, 8]. Though it is very important to identify the DEGs between normal and tumor tissues, it is also of great value to study the DEGs between tumor tissues from LTSs and short-term survivors (STSs, survival ≤ 1 years) of HCC [3], because these genes may be critical determinants for the survival time of patients or potential biomarkers for prognostic prediction and therapy. However, such genes have not yet been identified.

Uncontrolled cellular proliferation is a hallmark of tumorigenesis and cancer progression [9]. Cellular proliferation is dynamically and strictly controlled during different phases of the cell cycle, especially the G_1_/S and G_2_/M transitions [9]. The G_1_/S transition is the most important breaker in the cell cycle, and after that period, extracellular stimulants are no longer required [10, 11]. This transition is tightly regulated by multiple genes and pathways, and its disruption is associated with cancer development [12, 13]. For example, cyclin D1 *(CCND1)*, a promoter of the G_1_/S transition, is overexpressed in estrogen receptor-positive breast cancer cells [14]. The G_1_/S transition is considered as an effective target for cancer therapies; for instance, palbociclib, a selective cyclin-dependent kinase 4/6 (CDK4/6) inhibitor that leads to G_1_/S arrest, was found to significantly prolong the progression-free survival of patients with hormone-receptor-positive metastatic breast cancer [15, 16]. Although the G_1_/S transition is very important in tumor development and therapy, its roles on HCC have not been clearly elucidated.

In this study, we investigated novel molecular biomarkers of HCC to provide effective prognostic predictors and therapeutic targets. For this purpose, we performed a combinational analysis of whole-exome sequencing data, whole-transcriptome sequencing data and patients’ clinical information from the Liver Hepatocellular Carcinoma (LIHC) project in The Cancer Genome Atlas (TCGA) database and the LIRI-JP project in the International Cancer Genome Consortium (ICGC) database. We then conducted *in vitro* and *in vivo* experiments to validate the reliability of these biomarkers in HCC.

## RESULTS

### The molecular differences between HCC tissues from LTSs and STSs

**Figure 1:**
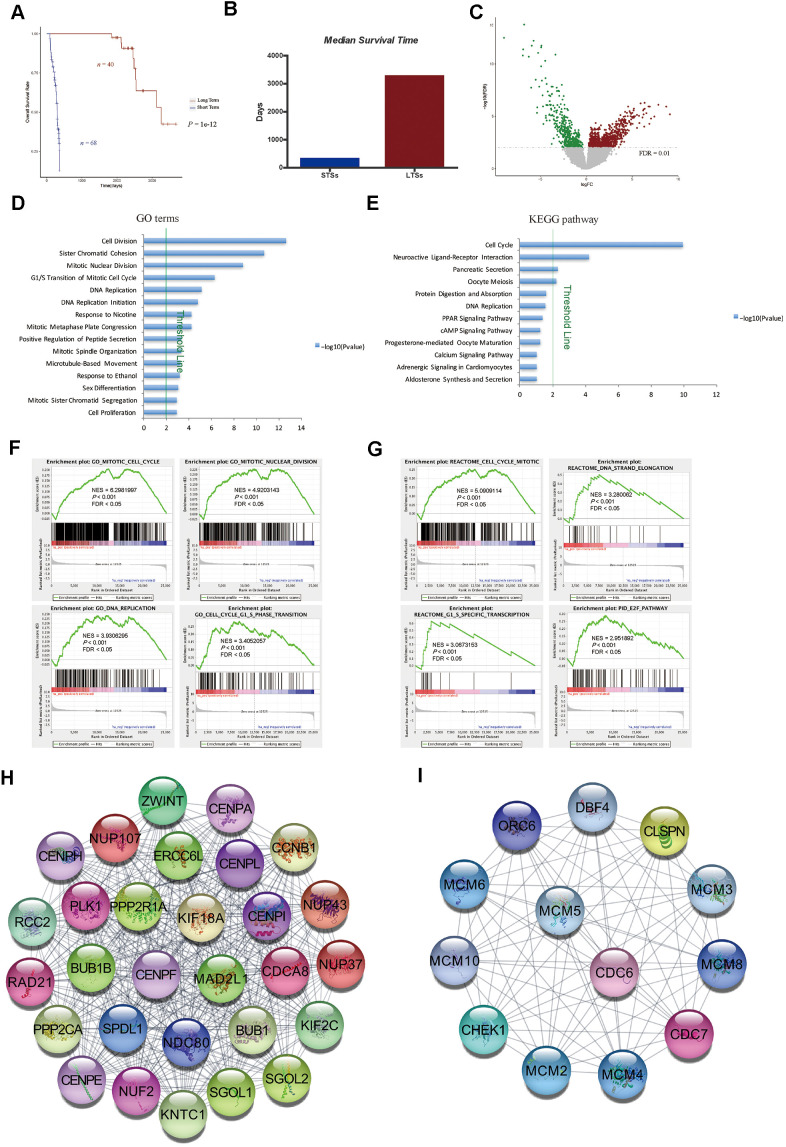
**Gene expression profiling of LTSs and STSs of HCC.** (**A**) Kaplan-Meier curve and log-rank test of STSs and LTSs of HCC. (**B**) The median survival days of STSs and LTSs of HCC. (**C**) Volcano plot of DEGs between 68 STS HCC samples and 40 LTS HCC samples. Red dots represent upregulated genes and green dots represent downregulated genes in HCC samples from STSs. (**D**) and (**E**) GO analysis (**D**) and KEGG pathway analysis (**E**) of DEGs via DAVID. Blue bars that cross the threshold line (*P* < 0.01) represent GO terms or KEGG pathways that differed significantly between the 68 STS HCC samples and the 40 LTS HCC samples. (**F**) and (**G**) GSEA of the global gene expression profiles of 68 STS HCC samples and 40 LTS HCC samples. Gene sets annotated with GO terms (**F**) and Gene sets annotated with canonical pathways mainly from Reactome and PID databases (**G**) were used in the analysis. NES = normalized enrichment score; *P* = nominal *P* value; FDR = false discovery rate. (**H**) and (**I**) The MCODE algorithm was used to identify the most significant modules in the PPI network constructed from the upregulated genes in STS HCC samples. The MCODE score of module 1 was 29 (**H**). The MCODE score of module 2 was 13 (**I**).

In this study, we first compared the global gene expression profiles of treatment-naive, surgically resected HCC samples from LTSs (n = 40; TCGA-LIHC cohort) and STSs (n = 68; TCGA-LIHC cohort) (Figure 1A and Table 1). The median survival time was much shorter in STSs than that in LTSs (Figure 1B). Based on our predefined cut-off criterion (false discovery rate [FDR] < 0.01), 1199 DEGs were identified, including 760 upregulated and 439 downregulated genes in STS tumor tissues versus LTS tumor tissues (Figure 1C).

**Table 1:** Clinical and pathological characteristics of patients in TCGA-LIHC project.

**Variable**	**Short term (n = 68) n (%)**	**Long term (n = 40) n (%)**
**Gender**		
Male	52 (76.5)	26 (65)
Female	16 (23.5)	14 (35)
**Age**		
Mean (Range)	59 (18-81)	59 (32-78)
**Race**		
Asian	42 (61.8)	18 (45)
White	22 (32.4)	20 (50)
Black or African American	1 (1.5)	1 (2.5)
NA	3 (4.4)	1 (2.5)
**Pathological Stage**		
I	22 (32.4)	23 (57.5)
II	15 (22.1)	9 (22.5)
III	27 (39.7)	7 (17.5)
IV	1 (1.5)	0 (0)
NA	3 (4.4)	1 (2.5)
**T Stage**		
T1	21 (30.9)	24 (60)
T2	15 (22.1)	9 (22.5)
T3	26 (38.2)	7 (17.5)
T4	5 (7.4)	0 (0)
NA	1 (1.5)	0 (0)
**N Stage**		
N0	48 (70.6)	34 (85)
N1	19 (27.9)	6 (15)
NX	1 (1.5)	0 (0)
**M Stage**		
M0	49 (72.0)	35 (87.5)
M1	1 (1.5)	0 (0)
MX	18 (26.5)	5 (12.5)
**Histologic Grade**		
G1	10 (14.7)	8 (20.0)
G2	39 (57.4)	21 (52.5)
G3	16 (23.5)	10 (25.0)
G4	2 (3.0)	0 (0)
NA	1 (1.5)	1 (2.5)

NA, Not Available.

We then used the Database for Annotation, Visualization and Integrated Discovery (DAVID) to perform Gene Ontology (GO) and Kyoto Encyclopedia of Genes and Genomes (KEGG) pathway enrichment analyses of the DEGs. We found that the cell cycle was the most significant pathways enriched in STS tumor samples (Figure 1D, 1E). Gene Set Enrichment Analysis (GSEA) using GO terms as the reference gene set revealed that GO terms associated with cell cycle regulation (e.g., mitotic cell cycle, mitotic nuclear division, DNA replication, cell cycle G_1_/S phase transition) were enriched in primary HCC samples from STSs compared with those from LTSs (Figure 1F). We then performed GSEA using canonical pathways mainly from Reactome and the Pathway Interaction Database (PID) as the reference gene set. The results also demonstrated that cell cycle-related pathways such as the mitotic cell cycle, DNA strand elongation, G_1_/S-specific transcription and E2F pathway were significantly enriched in tumor tissues from STSs (Figure 1G).

Metastasis and the anticancer immune response are very important determinants of the treatment response and prognosis of HCC patients [17, 18]. Therefore, we assessed the expression of genes involved in these processes in HCC samples from STSs and LTSs. Surprisingly, no obvious differences were observed between the two groups (Supplementary Figure 1), suggesting that these pathways do not critically influence the survival of HCC patients.

To confirm the correlation between cell cycle-related gene expression and the survival of HCC patients, we constructed a protein-protein interaction (PPI) network with the upregulated genes in STS HCC samples, and used the Molecular Complex Detection (MCODE) algorithm to determine the top significant modules in the network. Consistently, many nodes in module 1 were target genes of E2F transcription factors (e.g., *BUB1B*, *CDCA8*, *CENPE*, *KIF2C*, *NUP107*, *PLK1* and *RAD21*) (Figure 1H), which promote the G_1_/S transition in the cell cycle via the CyclinD-CDK4/6-Retinoblastoma-E2F pathway. In addition, many nodes in module 1 were cell cycle regulators (e.g., *BUB1* and *CCNB1*) (Figure 1H). Most of the nodes in module 2 were also target genes of E2Fs (e.g., *MCM2-6*, *ORC6* and *CHEK1*) (Figure 1I). These data suggested that genes involved in cell cycle regulation, but not in cancer metastasis or antitumor immunity, are essential determinants of the survival time of HCC patients.

### Cell cycle pathways were greatly enriched in matched tumor tissues from STSs of HCC

**Figure 2:**
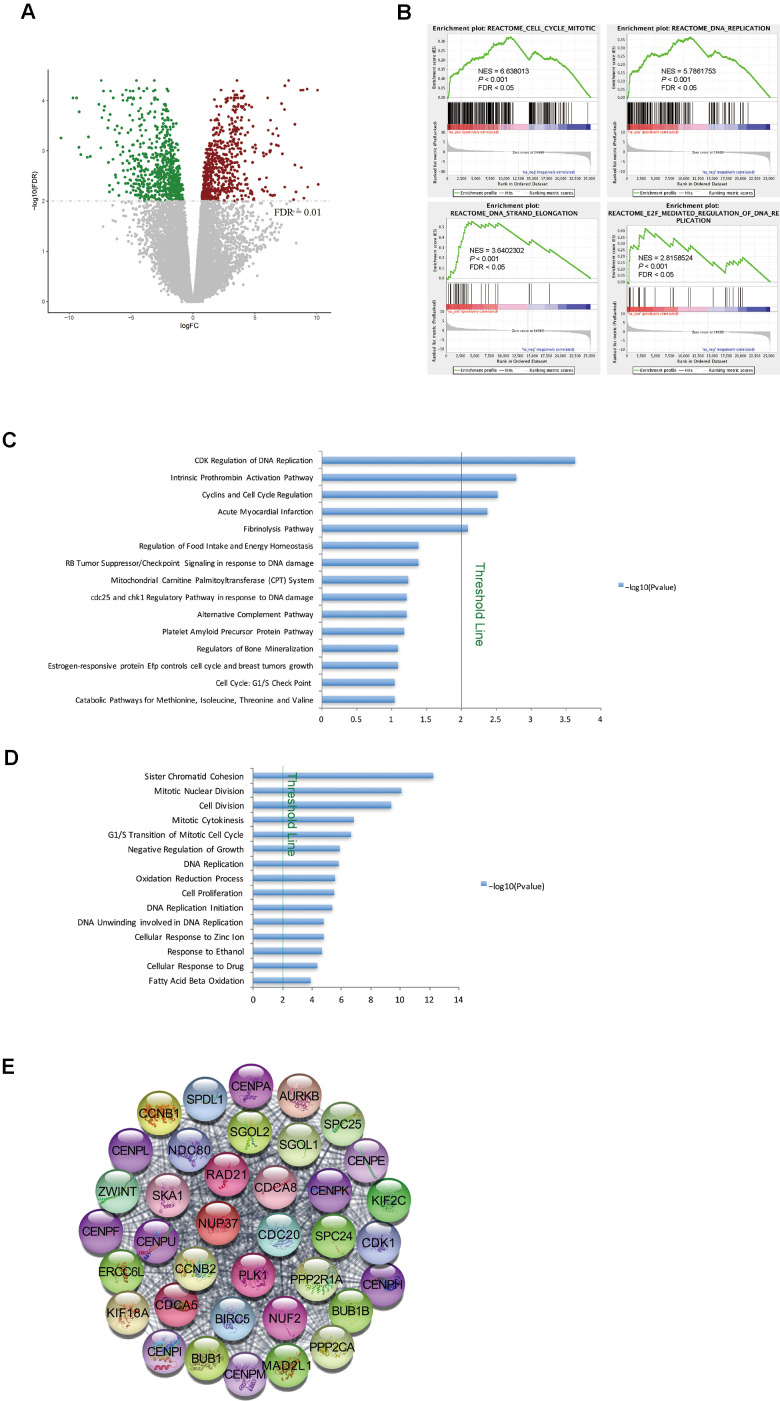
**Cell cycle pathways were enriched in tumor samples from STSs.** (**A**) Volcano plot of the DEGs between primary tumor tissues and matched normal liver tissues from nine STSs. Red dots represent upregulated genes and green dots represent downregulated genes in the primary tumor tissues. (**B**) GSEA of the global gene expression profiles of nine primary tumor tissues and matched normal liver tissues from STSs. Gene sets were annotated with canonical pathways mainly from Reactome. NES = normalized enrichment score; *P* = nominal *P* value; FDR = false discovery rate. (**C**) and (**D**) BioCarta pathway analysis (**C**) and GO analysis (**D**) of DEGs via DAVID. Blue bars that cross the threshold line (*P* < 0.01) represent pathways that differed significantly between primary tumor tissues and matched normal liver tissues from nine STSs. (**E**) The MCODE algorithm was used to identify the most significant module in the PPI network constructed from the upregulated genes in STS HCC samples. The MCODE score of this module was 37.

Matched primary tumor tissues and normal liver tissues were available for nine of the STSs included in this study. Therefore, we analyzed the global gene expression profiles of these tissues. After filtering the data based on a predefined cut-off criterion (FDR < 0.01), we identified 1356 DEGs between the tumor and normal tissues of STSs, including 668 upregulated and 688 downregulated genes in the tumor tissues versus normal liver tissues (Figure 2A). GSEA revealed that gene sets involved in cell cycle regulation (e.g., the mitotic cell cycle, DNA replication and E2F-mediated regulation of DNA replication) were enriched in the primary tumor tissues (Figure 2B).

Next, we used DAVID for GO and BioCarta pathway analyses of the DEGs between tumor and normal tissues from STSs. The BioCarta pathway analysis indicated that CDKs and cyclins, which regulate DNA replication and the cell cycle, were overrepresented in STS tumor samples (Figure 2C). In the GO analysis, cell cycle-related terms such as mitotic nuclear division and the G_1_/S transition of the mitotic cell cycle were also significantly upregulated in STS tumor samples (Figure 2D).

We then constructed a PPI network with the upregulated genes in STS HCC samples, and used MCODE algorithm to identify the most significant module (Figure 2E). Many nodes in this module were target genes of E2Fs (e.g., *CDK1*, *BUB1B*, *CDCA8*, *CDC20*, *CCNB2*, *CENPE*, *PLK1* and *RAD21*) (Figure 2E). These findings demonstrated that genes involved in cell cycle pathways were upregulated in tumor tissues from STSs.

### The upregulation of cell cycle pathways was associated with a poor prognosis in HCC patients

**Figure 3:**
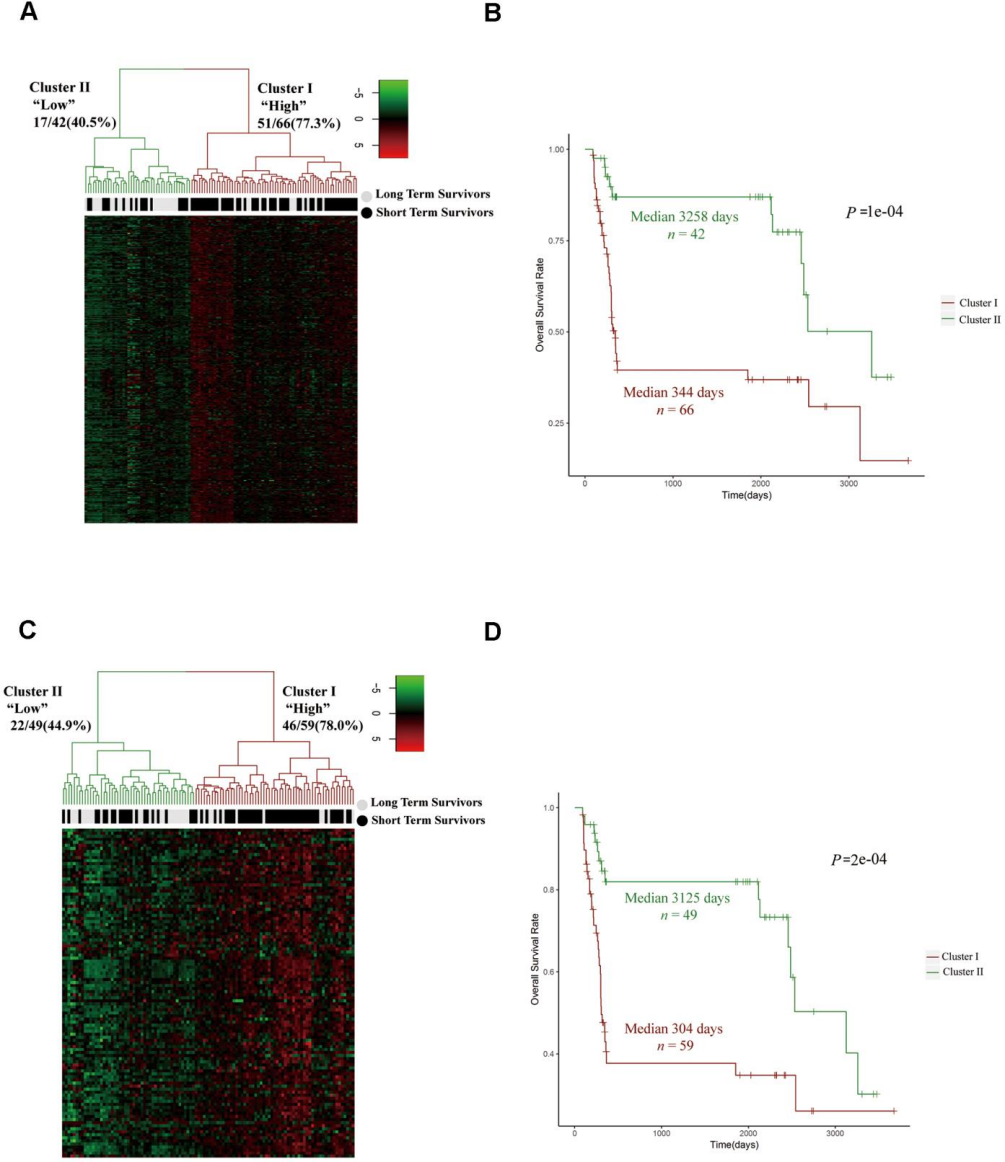
**The upregulation of cell cycle pathways was associated with a poor prognosis in HCC patients.** (**A**, **C**) Unsupervised hierarchical clustering with Euclidean distances and Ward linkages of the expression matrices of 300 mitotic cell cycle genes in Reactome (**A**) and 108 cell cycle G_1_/S phase transition genes in the GO resource (**C**) for 108 tumor samples (68 STS and 40 LTS). Rows indicate the genes and columns indicate the patients. The patient survival status for each tumor is depicted directly above each column. Cluster I expressed higher levels of the genes in these two pathways, while cluster II expressed lower levels. (**B**) Kaplan-Meier curves for the clusters resulting from the unsupervised hierarchical clustering in (**A**). (**D**) Kaplan-Meier curves for the clusters resulting from the unsupervised hierarchical clustering in (**C**).

To further investigate the relationship between the expression of cell cycle-related genes and the prognosis of HCC patients, we analyzed the expression levels of 300 mitotic cell cycle genes from the Reactome in 40 LTS and 68 STS tumor samples. After stratifying the tumor samples based on their gene expression patterns and examining the proportion of LTSs, we observed two clusters. Cluster I expressed higher levels of mitotic cell cycle genes and captured a higher proportion of STSs (77.3%), while cluster II expressed lower levels of mitotic cell cycle genes and captured a lower proportion of STSs (40.5%) (Figure 3A). Then, we examined the prognoses of the patients in these clusters, and found that cluster I exhibited a poorer survival probability, whereas cluster II exhibited a better prognosis (Figure 3B).

To confirm these results, we stratified the tumor samples from LTSs and STSs into two clusters based on their expression of cell cycle G_1_/S phase transition genes from the GO resource. Cluster I expressed higher levels of cell cycle G_1_/S phase transition genes and captured a higher proportion of STSs (78.0%), while cluster II expressed lower levels of cell cycle G_1_/S phase transition genes and captured a lower proportion of STSs (44.9%) (Figure 3C). Moreover, cluster I exhibited a poorer prognosis (Figure 3D). These data suggested that the upregulation of cell cycle pathways is associated with a poorer prognosis in HCC patients.

### The expression signature of G_1_/S phase transition inducers was an independent prognostic factor in HCC patients

**Figure 4:**
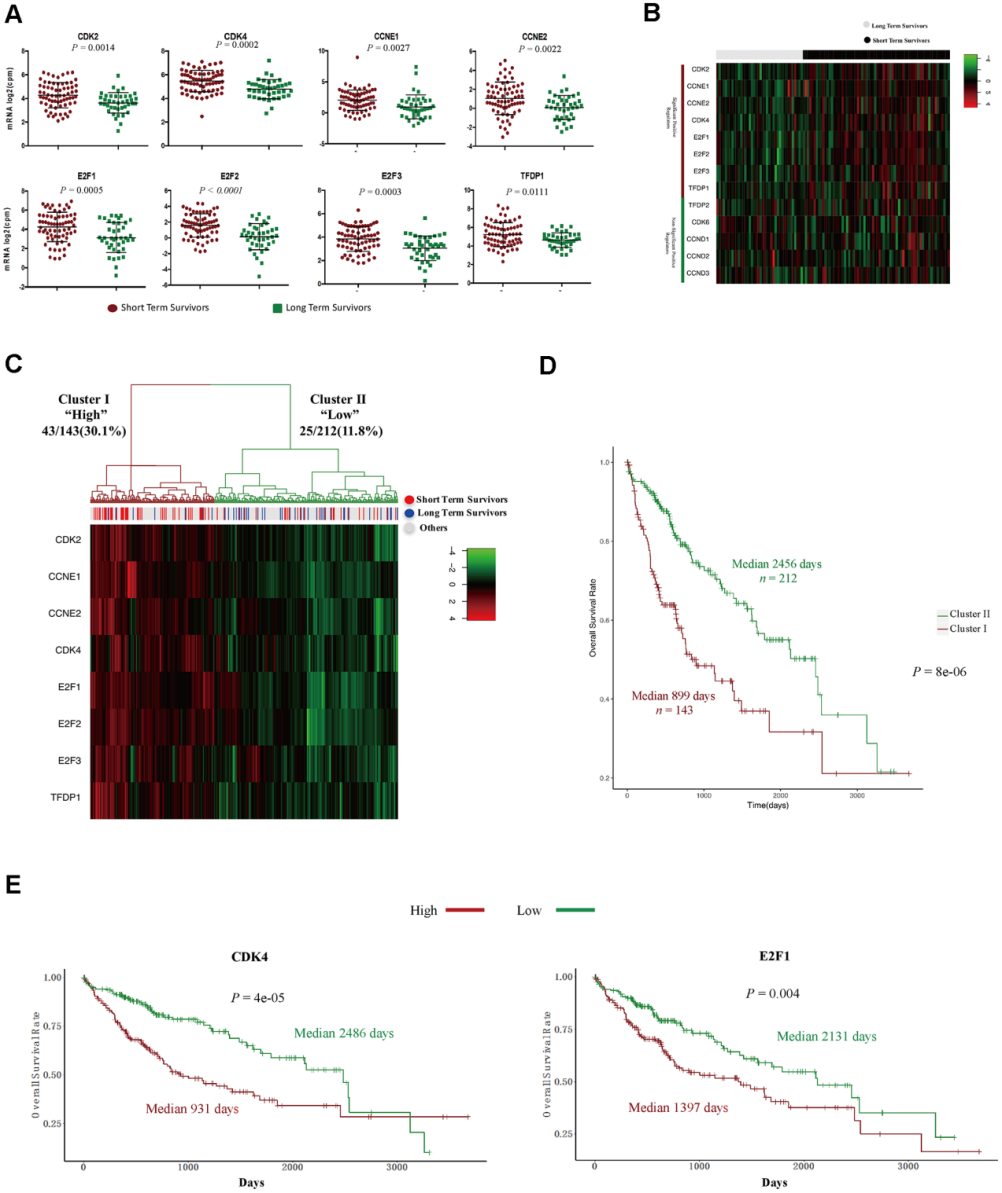
**The expression signature of eight positive regulators of the G**_1_**/S cell cycle transition was an independent prognostic factor in HCC.** (**A**) Tumor sample transcriptomic profiling of eight positive regulators of the G_1_/S cell cycle transition that differed significantly between STSs and LTSs of HCC. The data are shown as the mean ± standard deviation, and were compared using an unpaired two-tailed Student’s t-test. cpm, counts per million. (**B**) Tumor sample expression signature heatmap of thirteen positive regulators of the G_1_/S cell cycle transition in STSs and LTSs of HCC. Rows indicate the genes and columns indicate the patients. The patient survival status for each tumor is depicted directly above each column. (**C**) Unsupervised hierarchical clustering with Euclidean distances and Ward linkages of the expression matrix of eight positive regulators of the G_1_/S cell cycle transition in 355 HCC samples. Rows indicate the genes and columns indicate the patients. The patient survival status for each tumor is depicted directly above each column. (**D**) Kaplan-Meier curves for the clusters resulting from the unsupervised hierarchical clustering in (**C**). (**E**) Kaplan-Meier curve and log-rank test for HCC patients based on the expression of CDK2 and E2F1.

*CDK4*, *CDK6* and *CDK2* are major inducers of the G_1_/S phase transition, and function in complexes with their cyclin partners [19]. In total, 13 positive regulators of the CyclinD-CDK4/6-Retinoblastoma-E2F pathway and the CyclinE-CDK2 pathway directly promote the cell cycle transition from G_1_ into S phase [19]. Therefore, we compared the expression of these genes between STSs and LTSs. The levels of eight positive regulators (*CDK2*, *CDK4*, *CCNE1*, *CCNE2*, *E2F1*, *E2F2*, *E2F3* and *TFDP1*) were significantly increased in STS than in LTS tumor samples (Figure 4A, 4B), whereas the levels of the other five positive regulators (*CDK6*, *CCND1*, *CCND2*, *CCND3* and *TFDP2*) did not differ between the two groups (Supplementary Figure 2). Then, we performed an unsupervised hierarchical clustering analysis to determine the expression patterns of these eight positive regulators in 355 HCC samples from TCGA. Two groups were observed: cluster I expressed higher levels of these genes, had a higher proportion of STSs (30.1%) and exhibited significantly poorer survival (Figure 4C, 4D), whereas cluster II expressed lower levels of these genes, had a lower proportion of STSs (11.8%) and exhibited a better prognosis (Figure 4C, 4D).

We then performed Cox proportional hazards regression analyses to investigate whether the expression pattern of these eight positive regulators or patients’ clinical characteristics were associated with overall survival. In a univariate analysis, five factors (residual tumor, pathologic stage, T stage, treatment procedures and the expression pattern of the eight positive regulators) were associated with patients’ overall survival with *P* values < 0.05 (Table 2). However, in a multivariate analysis, only R2 residual tumors (hazard ratio = 9.5270, *P* = 0.03652) and high levels of the eight positive regulators (hazard ratio = 1.8178, *P* = 0.00478) were found to be independent prognostic factors in HCC patients (Table 2). Subsequently, we examined the effects of each of the eight G_1_/S phase transition inducers on patients’ prognoses. Notably, higher levels of seven of these genes (*CDK2*, *CDK4*, *CCNE1*, *CCNE2*, *E2F1*, *E2F2* and *E2F3*) were associated with poorer survival (Figure 4E and Supplementary Figure 3). Thus, these cell cycle-related genes that promote the G_1_/S transition are promising prognostic markers in HCC patients.

**Table 2:** Univariate and multivariate cox proportional hazard regression analysis of 355 HCC patients in TCGA-LIHC project.

**Variables**	**Univariate cox regression analysis**			**Multivariate cox regression analysis**		
	**P**	**HR**	**95%CI**	**P**	**HR**	**95%CI**
Age (<*60 vs. ≥60)*	0.283	1.212	0.8534 to 1.721			
Residual Tumor						
R0						
R1	0.2564	1.517	0.7388 to 3.114	0.94781	0.9660	0.3425 to 2.725
R2	0.0167	11.461	1.5565 to 84.399	0.03652	9.5270	1.1519 to 78.796
Pathologic Stage (^I^*+*^I^ *vs.*^I^ *+*^IV^*)*	3.3e-06	2.42	1.667 to 3.511	0.85305	1.2103	0.1606 to 9.118
Cluster (Low vs. High)	1.21e-05	2.184	1.539 to 3.098	0.00478	1.8178	1.2002 to 2.753
T Stage (1,2 vs. 3,4)	3.37e-07	2.51	1.762 to 3.574	0.65792	1.5751	0.2108 to 11.769
Gender (Female vs. Male)	0.29	0.8252	0.5778 to 1.178			
Histologic Grade						
G1						
G2	0.525	1.186	0.7003 to 2.010			
G3	0.492	1.213	0.6990 to 2.105			
G4	0.539	1.406	0.4737 to 4.174			
Procedure						
Extended Lobectomy						
Lobectomy	0.68217	0.8789	0.4739 to 1.6301	0.64648	1.1766	0.5873 to 2.357
Segmentectomy, Multiple	0.28649	0.7040	0.3692 to 1.3424	0.81762	1.0903	0.5229 to 2.273
Segmentectomy, Single	0.00317	0.3290	0.1572 to 0.6884	0.06069	0.4389	0.1856 to 1.038
Other^*^	0.11096	1.8678	0.8663 to 4.0269	0.37462	1.6500	0.5462 to 4.984

HR, Hazard Ratio. * Other procedures include hepatic resection, left hepatic lobectomy, total hepatectomy with transplant and so on.

### HCC patients with *TP53* mutations tended to be STSs

**Figure 5:**
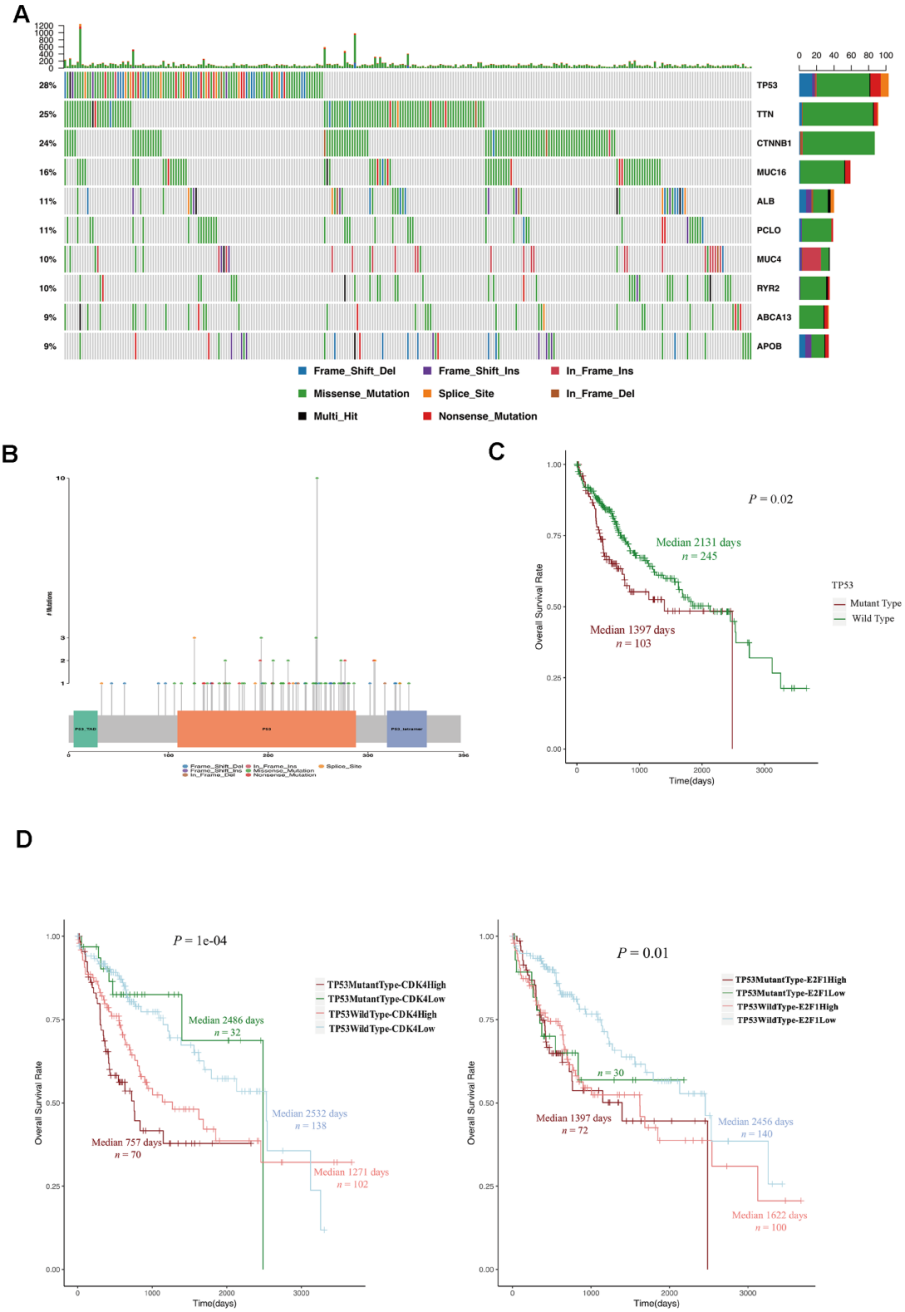
**TP53 mutations were associated with a poor prognosis in HCC patients.** (**A**) OncoPlot of the top ten mutated genes. The upper bar plot indicates the number of genetic mutations per patient, while the right bar plot displays the number of genetic mutations per gene. The mutation types were added as annotations on the bottom. Variants annotated as Multi_Hit are genes that mutated more than once in the same sample. (**B**) The lollipopPlot of *TP53*. The amino acid axis was labeled for the domain. The mutation types were added as annotations on the bottom. (**C**) Kaplan-Meier curve and log-rank test for HCC patients based on the *TP53* mutational status classification. (**D**) Kaplan-Meier curve and log-rank test for HCC patients based on the TP53 mutational status and the expression of CDK2 and E2F1. The patients were stratified into the high expression group and the low expression group according to the median of normalized RNA-seq data. The patients were stratified into mutant TP53 group and wild type TP53 group according to TP53 mutational status.

Next, we investigated whether there were differences in somatic mutations between LTSs and STSs from TCGA. TP53 is a well-known negative regulator of the G_1_/S phase transition of the cell cycle [20–22]. We found that *TP53* mutations accounted for more than 28% of the mutations in HCC patients, and the majority of *TP53* mutations were missense mutations (Figure 5A, 5B). Interestingly, HCC patients with *TP53* mutations mainly belonged to the STS group (Table 3) and exhibited a poor overall survival rate (Figure 5C). HCC patients with *TP53* mutations and high *CDK4* (or *E2F1*) levels had the shortest survival expectancy of all the groups (Figure 5D). These data suggested that *TP53* mutations contribute to and are promising diagnostic markers of STSs.

**Table 3:** The relationship between TP53 mutational status and survival status in 348 HCC patients in TCGA-LIHC project.

**Survival status**	**TP53 mutant type**	**TP53 wild type**	**P value**
STSsN (%)	26 (25.3)	38 (15.5)	0.01891*
LTSsN (%)	6 (5.8)	34 (13.9)	
Other N (%)	71 (68.9)	173 (70.6)	

* Fisher’s Exact Test. STSs, Short Term Survivors. LTSs, Long Term Survivors.

### The expression of G_1_/S phase transition inducers and *TP53* mutations predicted survival in the ICGC dataset

**Figure 6:**
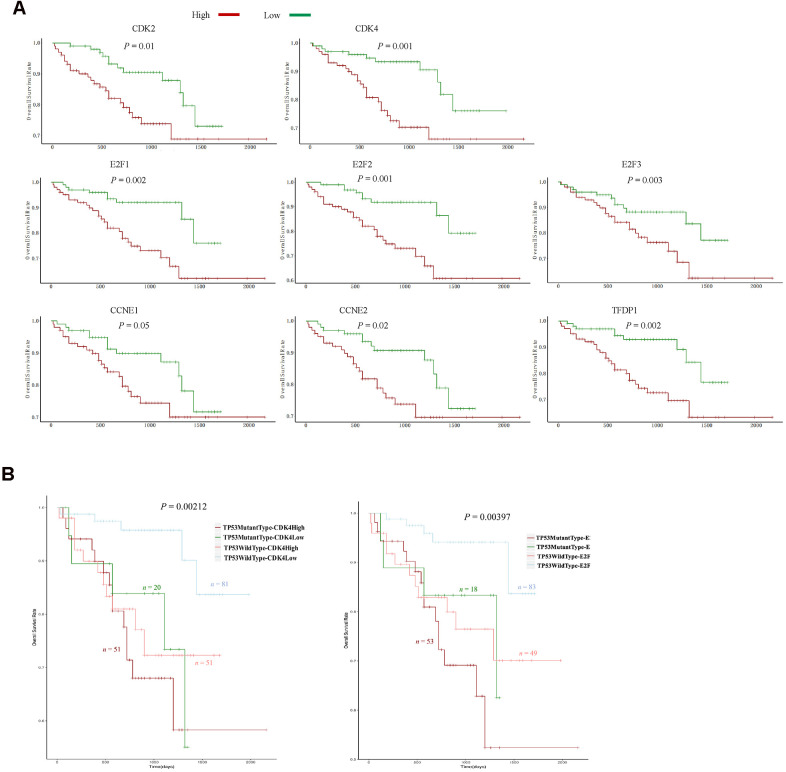
**The expression of eight G_1_/S phase transition inducers and TP53 mutations predicted survival in the ICGC dataset.** (**A**) Kaplan-Meier curve and log-rank test for HCC patients based on the expression of the eight G_1_/S phase transition inducers. (**B**) Kaplan-Meier curve and log-rank test for HCC patients based on the *TP53* mutational status and the expression of *CDK2* and *E2F1*. The patients were stratified into the high expression group and the low expression group according to the median of normalized RNA-seq data. The patients were stratified into mutant TP53 group and wild type TP53 group according to TP53 mutational status.

We then investigated whether the eight positive regulators of the G_1_/S phase transition and *TP53* mutations exhibited similar prognostic trends in other HCC datasets. We chose the LIRI-JP project in the ICGC database, which contained data from 203 HCC patients. Consistent with our previous results (Figure 4E and Supplementary Figure 3), higher levels of seven inducers of the G_1_/S phase transition (*CDK2*, *CDK4*, *CCNE2*, *E2F1*, *E2F2*, *E2F3* and *TFDP1*) were associated with poorer survival (Figure 6A). Moreover, HCC patients with wild-type *TP53* and low *CDK4* (or *E2F1*) expression had the longest survival expectancy (Figure 6B). These data confirmed that the levels of seven G_1_/S phase transition inducers and *TP53* mutations predicted survival in HCC patients.

### Abemaciclib significantly inhibited the proliferation of HCC cells *in vitro*

**Figure 7:**
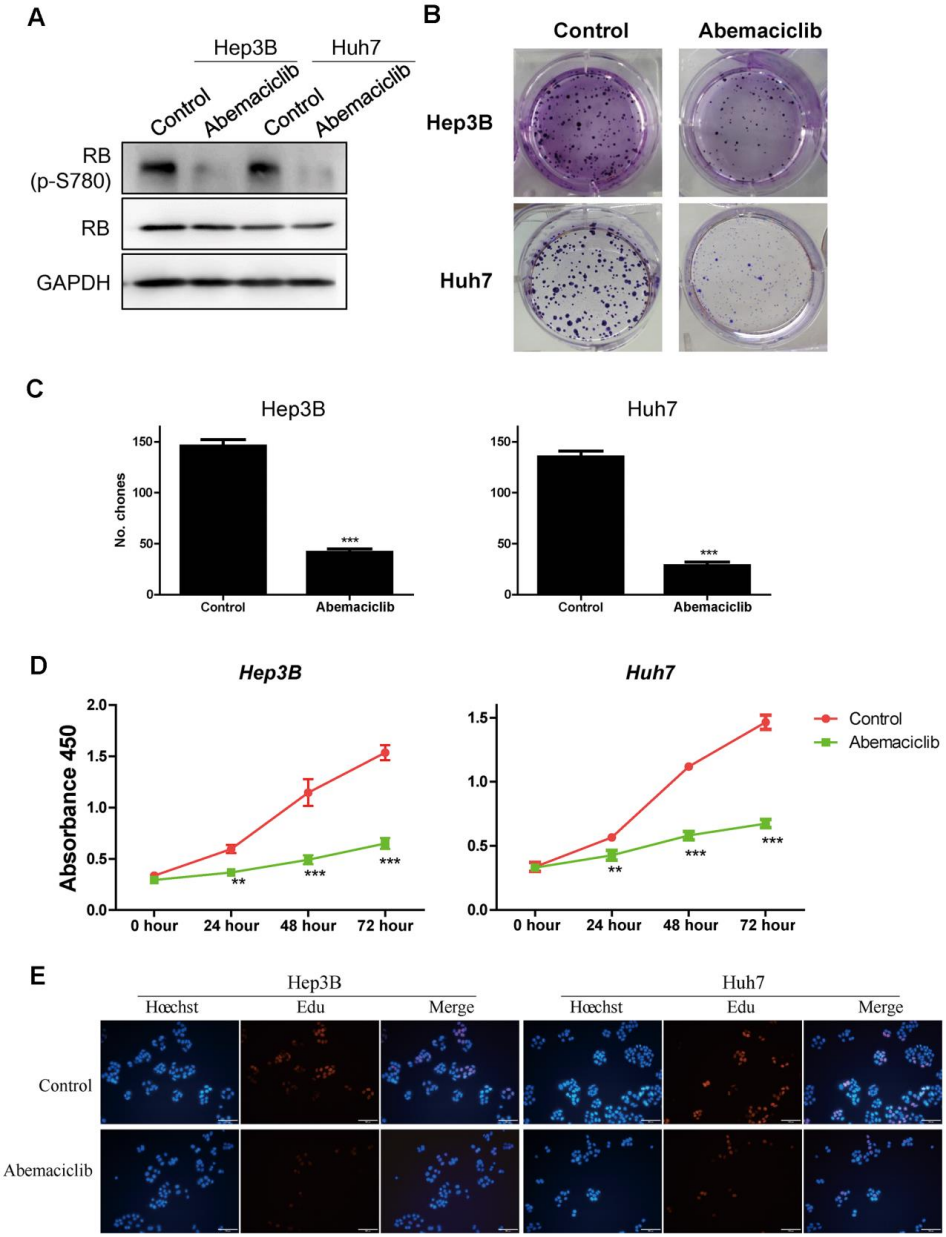
**Abemaciclib substantially inhibited the proliferation of HCC cells *in vitro*.** (**A**) The protein levels of retinoblastoma (p-S780) and retinoblastoma in Hep3B and Huh7 cells. (**B**, **C**) Clone formation assays were used to detect the effects of abemaciclib on the proliferation of Hep3B and Huh7 cells. (**D**) A CCK-8 assay was used to analyze cell proliferation in control and abemaciclib-treated Hep3B and Huh7 cells. (**E**) An Edu assay was used to analyze cell proliferation in control and abemaciclib-treated Hep3B and Huh7 cells. Bar= 100μm; For (**C**, **D**), * was compared with Control (**, P < 0.01; ***, P < 0.001).

Because higher levels of *CDK4* and its downstream effector *E2F1* were observed in STSs (Figure 4E and Figure 6A), we speculated that inhibiting CDK4 might prolong the survival of HCC patients. Therefore, we assessed the effects of abemaciclib (the first FDA-approved CDK4 inhibitor for breast cancer treatment) on the HCC cell lines Hep3B and Huh7 [23]. Retinoblastoma (RB) is inactivated upon its phosphorylation by CDK4, so we detected the protein levels of phosphorylated RB and total RB in these two cell lines. While total RB protein levels remained relatively constant in Hep3B and Huh7 cells, phospho-S780-RB levels decreased significantly following abemaciclib treatment (Figure 7A). Abemaciclib also notably reduced the clone numbers of these two cell lines (Figure 7B, 7C). A Cell Counting Kit 8 (CCK-8) assay (Figure 7D) and a 5-ethynyl-2´-deoxyuridine (Edu) assay (Figure 7E) confirmed that abemaciclib repressed HCC cell proliferation. These data demonstrated that abemaciclib substantially inhibited the proliferation of HCC cells *in vitro*.

### Abemaciclib inhibited the tumor growth of HCC cells *in vivo*

**Figure 8:**
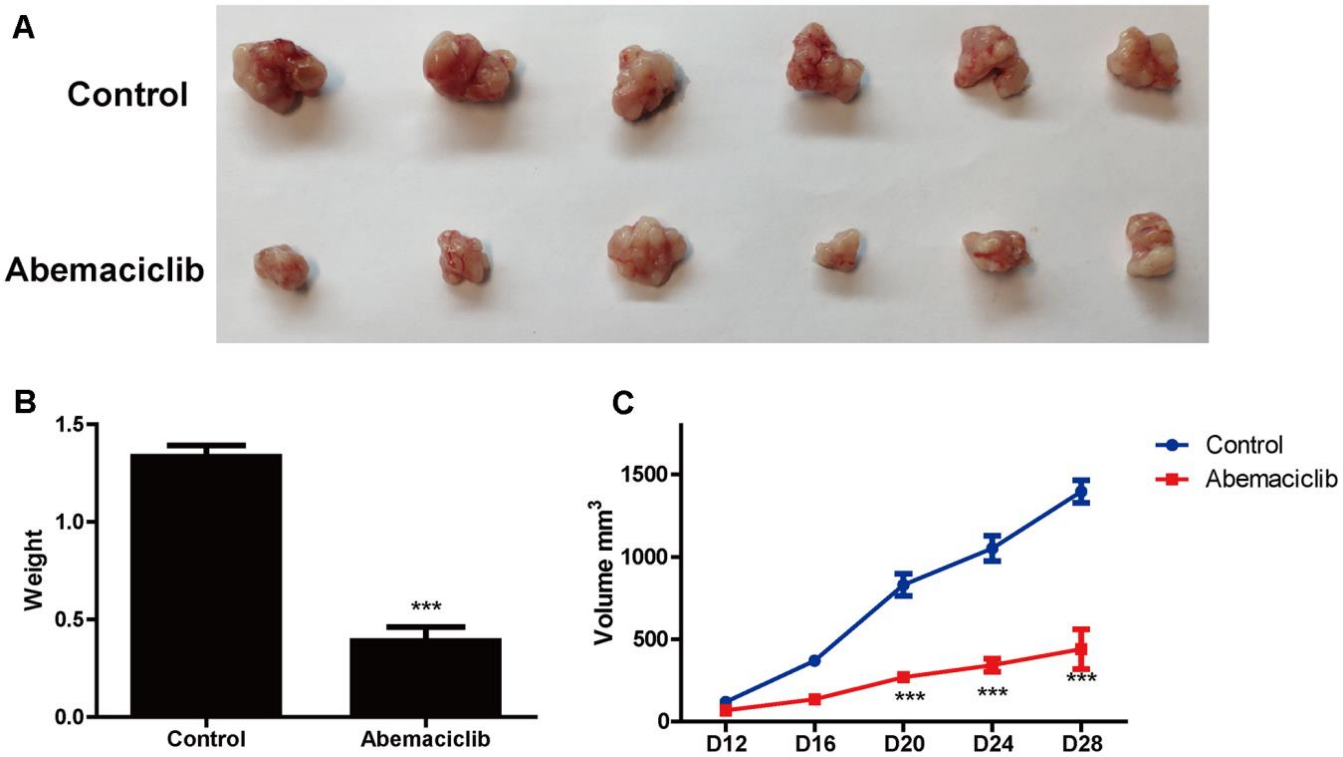
**Abemaciclib substantially inhibited the proliferation of HCC cells *in vivo*.** (**A**) Phenotypes of tumors derived from Hep3B cells in mice treated with the vehicle (DMSO) or abemaciclib. (**B**) The weights of Hep3B cell tumors were detected in mice treated with the vehicle or abemaciclib. (**C**) The dynamics of tumor development in mice treated with the vehicle or abemaciclib. The tumor volumes were measured as follows: Volume = length*width*width/2. Measurements were taken at the indicated time points. For (**B**, **C**), * was compared with Control (***, P < 0.001).

Finally, we assessed the effects of abemaciclib on HCC development *in vivo* in mice subcutaneously injected with Hep3B cells. Abemaciclib treatment significantly reduced the size (Figure 8A) and weight (Figure 8B) of the tumors in these mice. Dynamic analyses of tumor development also revealed that the tumor volume was significantly reduced after abemaciclib treatment (Figure 8C). These data demonstrated that abemaciclib can significantly inhibit the proliferation of HCC cells *in vivo*.

## DISCUSSION

Prognostic prediction for cancer patients is vital, not only for identifying potential STSs who will need special treatments, but also for designing appropriate strategies to prolong survival [24]. For example, B-Raf proto-oncogene (BRAF) mutation, especially V600E, is an ideal marker of poor prognosis and new treatment target in colon cancer patients [25, 26]. However, the ability to predict HCC patients’ prognoses is still limited [1]. Here, we identified molecular markers that could effectively distinguish STSs from LTSs of HCC, allowing HCC patients to be stratified into good and poor prognosis groups easily and efficiently. Patients with low levels of eight G_1_/S phase transition inducers survived for nearly 7 years, whereas patients with high levels of these eight genes had a survival expectancy of only 2.07 years. Besides G_1_/S phase transition inducers, *TP53* mutations are another critical factors to STSs, and promising biomarker for HCC prognosis. Therefore, we speculated that HCC patients with heightened expression of G_1_/S phase transition inducers and *TP53* mutations tent to be STSs; and these predicted poor prognosis patients desperately need to receive more timely and effective treatments.

Many proteins involved in the G_1_/S transition contribute substantially to the development and progression of HCC [27–30]. Aberrant expression and activation of these genes can stimulate the proliferation of HCC cells [31, 32], and uncontrolled cell proliferation promotes cancer initiation [33]. Here, we demonstrated that genes that induce cell proliferation were highly expressed in STSs, suggesting that they are critical for cancer development. Sonntag et al. reported that *CCNE1* and *CDK2* were crucial for the initiation but not the progression of HCC [23]. These contradictory results may largely because of their synergistic effects; thus, it is important to treat these proteins as a unit. Intriguingly, though immune pathway activation and metastasis are key determinants of cancer development and patient survival [34, 35], we observed no significant differences in these two pathways between STSs and LTSs (Supplementary Figure 1). These interesting results may be contributed to the characteristics of HCC, including the short survival time and shortage of immunotherapies [18, 36]. Further clinical studies will be valuable to elucidate the involvement of these pathways in the development and treatment of HCC.

Aberrant activation of *CDK4,* one of the eight positive regulators of the G_1_/S transition, is frequently seen in various cancers, including HCC [37, 38]. As one of the most fatal cancers, HCC is an aggressive cancer with dismal prognosis, largely contributing to lack of efficient treatment [39]. In this study, we demonstrated that *CDK4* expression was significantly lower in LTS HCC tissues than in STS HCC tissues, suggesting that inhibiting CDK4 might prolong the survival of HCC patients. In recent years, abemaciclib, a highly selective and FDA-approved oral inhibitor of CDK4, has been clinically applied with promising therapeutic effects [40–42]. However, its effects on HCC have not been clarified [43]. We found that abemaciclib suppressed HCC cell proliferation *in vitro* and tumor growth *in vivo*, suggesting that it was an attractive drug for HCC patients, especially those with high *CDK4* expression. Thus, it is of value to investigate the effects of abemaciclib on HCC through further experimentations and clinical explorations. Additionally, Goel demonstrated that CDK4 inhibition activates anti-tumor immunity [44]; therefore it is of interest to treat STS patients with combined therapy including CDK4/6 inhibitor and immune-checkpoint inhibitor. Besides *CDK4*, *CDK2* is highly expressed in the tumor tissues of STSs. Accumulating evidence suggest that CDK2 inhibition are particularly useful for several cancers including lung cancer, prostate cancer, and breast cancer [45]. Herein, HCC patients with high CDK2 might have particular susceptibility to CDK2 inhibition.

Our study had several limitations. First, the numbers of LTSs and STSs in the TCGA-LIHC cohort were relatively small (40 LTSs and 68 STSs). Moreover, only nine matched HCC and normal liver samples from the STSs were available. Second, the clinical applicability of the CDK4/6 inhibitor abemaciclib for HCC treatment is still unclear, and clinical trials are needed to investigate its efficacy. Further studies are also needed to determine whether positive regulators of the G_1_/S transition can be used as prognostic predictors and treatment targets in HCC patients.

In summary, we performed a comprehensive comparison of the transcriptome between STSs and LTSs of HCC, and demonstrated that genes that induce the G_1_/S transition were significantly enriched in STSs. *TP53* mutations, the most common mutations in HCC, were also associated with STSs. Higher expression of eight G_1_/S transition inducers strongly predicted a poorer prognosis in HCC patients. In terms of HCC treatment, abemaciclib substantially inhibited the proliferation of H CC cells. This study has suggested new targets for the personalized diagnosis and treatment of STSs of HCC.

## MATERIALS AND METHODS

### TCGA data

RNA-seq raw counts (HTSeq - Counts) data, Mutation Annotation Format (MAF) data (generated by the mutect2 algorithm from whole-exome sequencing data), clinical data and survival data were downloaded from the LIHC project of TCGA (https://cancergenome.nih.gov/). LTSs were defined as patients whose overall survival extended 5 years from surgery, while STSs were defined as patients who survived > 3 months and < 1 year from surgery, to exclude perioperative mortalities. In total, 361 HCC patients with survival data were included in our analyses, including 40 LTSs and 68 STSs. RNA-seq data from primary tumor tissues were available for 355 of these HCC patients. RNA-seq data from primary tumor tissues and matched normal tissues were available for nine STSs. Mutation data were available for 348 HCC patients, including 342 patients with RNA-seq data from primary tumor tissues.

### ICGC data

Normalized RNA-seq data (fragments per kilobase million, FPKM), annotated mutation data, clinical data and survival data were downloaded from the LIRI-JP project of the ICGC database (https://www.icgc.org). In total, 203 HCC patients were included in our analyses. RNA-seq data from primary tumor tissues, along with mutation data and survival data, were available for all patients.

### Prognostic analyses

Survival curves were constructed using the Kaplan-Meier method and compared using the log-rank test via the survival R package. Univariate and multivariate Cox proportional hazards regression analyses were conducted using the survival R package.

### DEG analysis

The DEGs between primary tumor tissues from LTSs and STSs or between primary tumor tissues and matched normal liver tissues from STSs were analyzed with the edgeR R/Bioconductor package [46]. Genes with FDRs < 0.01 were considered to be significantly DEGs. GO, KEGG and BioCarta pathway enrichment analyses of the DEGs were performed using DAVID (https://david.ncifcrf.gov/) [47].

### PPI network analysis

We used the plug-in StringApp [48] in Cytoscape software [49, 50] to construct PPI network of the DEGs with default parameters. Subsequently, Molecular Complex Detection (MCODE) [51] in Cytoscape was applied to screen top significant modules within PPI network with default parameters.

### GSEA

GSEA was implemented through GSEAPreranked tool in GSEA software (http://www.broadinstitute.org/gsea/) [52]. The value of log2 (FC) calculated by edgeR package was used as ranking metric. We chose C5 collection that contains genes sets annotated by GO terms in the Molecular Signatures Database (MSigDB) (https://www.gsea-msigdb.org/gsea/msigdb) as the reference gene set in GSEA software. We also used the canonical pathways sub-collection of C2 collection in the MSigDB as the reference gene set in GSEA software. The annotated pathways in this sub-collection are mainly from BIOCARTA, KEGG, REACTOME and PID databases.

### Analysis and visualization of somatic mutations

We used the Maftools R/Bioconductor package [53] to extract detailed mutational information from the MAF file. The OncoPlot function [54] and the lollipopPlot function were used to create the OncoPlot of the top ten mutated genes and the lollipopPlot of *TP53*, respectively.

### Hierarchical clustering

The gene expression data for two genes sets (the cell cycle G_1_/S phase transition in GO, and the mitotic cell cycle in Reactome) were extracted from normalized and log2-transformed RNA-seq data from 108 HCC samples in TCGA. Unsupervised hierarchical clustering was used to discover groups based on the expression patterns of the genes in these two genes sets. In total, 108 cell cycle G_1_/S phase transition genes from the GO resource and 300 mitotic cell cycle genes from the Reactome were used. The expression values of these genes in the 108 samples were hierarchically clustered with Euclidean distances and Ward linkages using the dendextend R package [55] and the heatmap.2 function in the gplots R package. The proportions of STSs in the resulting clusters were calculated. Kaplan-Meier survival curves were plotted for the clusters and compared using the log-rank test.

Normalized and log2-transformed RNA-seq data for eight positive regulators of the G_1_/S phase transition (*CDK2*, *CDK4*, *CCNE1*, *CCNE2*, *E2F1*, *E2F2*, *E2F3* and *TFDP1*) in 355 HCC samples from TCGA were hierarchically clustered with Euclidean distances and Ward linkages using the dendextend R package [55] and the heatmap.2 function in the gplots R package. The proportions of STSs in the resulting clusters were calculated. Kaplan-Meier survival curves were plotted for the clusters and compared using the log-rank test.

### Cells and reagents

The HCC cells (Hep3B and Huh7) were obtain from the Cell Bank of the Type Culture Collection of the Chinese Academy of Sciences, Shanghai Institute of Cell Biology, Chinese Academy of Sciences. They were both cultured in high-glucose Dulbecco’s modified Eagle’s medium (Gibco; Thermo Fisher Scientific, Inc., Waltham, MA, USA) supplemented with 10% FBS (Gibco), and 1% penicillin/streptomycin (Hyclone; GE Healthcare Life Sciences, Logan, UT, USA). The HCC cells were cultured in a humidified incubator containing 5% CO_2_ at 37° C (Thermo Fisher Scientific, Inc.). Abemaciclib (S5716) was purchased from Selleck Chemicals (Houston, TX, USA). A stock solution of abemaciclib was prepared in dimethyl sulfoxide (DMSO; Sigma, St. Louis, MO, USA) and stored at - 80° C. Abemaciclib was diluted to a final concentration of 10 μM for use in experiments.

### Western blotting

HCC cells were lysed with a strong radioimmunoprecipitation assay buffer containing Halt^TM^ Protease Inhibitor Cocktail (Thermo, Waltham, MA, USA). The concentrations of the proteins in the lysate were determined with a bicinchoninic acid protein assay kit (Pierce, Rockford, IL, USA). Proteins were detected on a 12% sodium dodecyl sulfate-polyacrylamide gel electrophoresis and then transferred onto a polyvinylidene fluoride membrane. The membranes were incubated overnight at 4° C with primary antibodies for retinoblastoma (phospho S780) (ab47763, Abcam, Cambridge, UK), retinoblastoma (ab181616) and GAPDH (BM1623, Boster, Wuhan, China), and then were incubated with the appropriate horseradish peroxidase-conjugated secondary antibodies for 2 hours. The proteins were detected using an Amersham Imager 600 (GE Healthcare Life Sciences, Boston, MA, USA).

### Clone formation assay

For the clone formation assay, 1000 HCC cells were seeded in each well of a six-well plate and cultured in an incubator for more than 12 days. The medium was replaced every three days. Then, the cells were fixed with methanol for 20 min and stained with crystal violet. Pictures were taken with a digital camera (Nikon, Japan).

### CCK-8 assay

HCC cells were cultured in 96-well plates at a density of 1000 cells/well. After the cells had been cultured for 12, 36 or 60 hours, each well was treated with 10 μL of the assay reagent (Dojindo Molecular Technologies, Kumamoto, Japan), and the plate was returned to the incubator for 2 hours. Then, the absorbance at 450 nm was recorded with a SpectraMax M5 microplate reader (Thermo Fisher Scientific, Waltham, MA, USA).

### Animal model

Twelve male BALB/c nude mice (four weeks old) were obtained from SLAC Laboratory Animal Company (Shanghai, China). For tumor generation, the mice received a subcutaneous axillary injection of Hep3B cells (6×10^6^ cells/mouse) in the right forelimb. The mice were gavaged with abemaciclib (50 ug/g) daily, beginning seven days after the cell injection. The mice were sacrificed after 28 days, and their tumor weights were evaluated.

### Ethical approval and consent to participate

All the animal experiments were performed according to National Institutes of Health guide for the care and use of laboratory animals (NIH Publications No. 8023, revised 1978) and were approved by the Institutional Animal Care and Use Committee of Tongji University.

### Statistical analysis

All the data represented three independent experiments, with the data expressed as the mean ± SD. Differences between the Control and Abemaciclib groups were analyzed using Student's t-test in SPSS 17.0. A p-value less than 0.05 was considered significant.

### Availability of data and materials

All data analyzed during this study are included in this published article. Raw and processed data are stored in the laboratories are available upon reasonable request.

## Supplementary Material

Supplementary Figures
