# Supplementary material for: Molecular characterization of long-term survivors of hepatocellular carcinoma
Source: Aging (Albany NY). 2021 Mar 3;13(5):7517–37. doi: 10.18632/aging.202615 (PMC7993728; doi:10.18632/aging.202615)
Supplement: Supplementary Figures [file aging-13-202615-s001.pdf]

SUPPLEMENTARY FIGURES

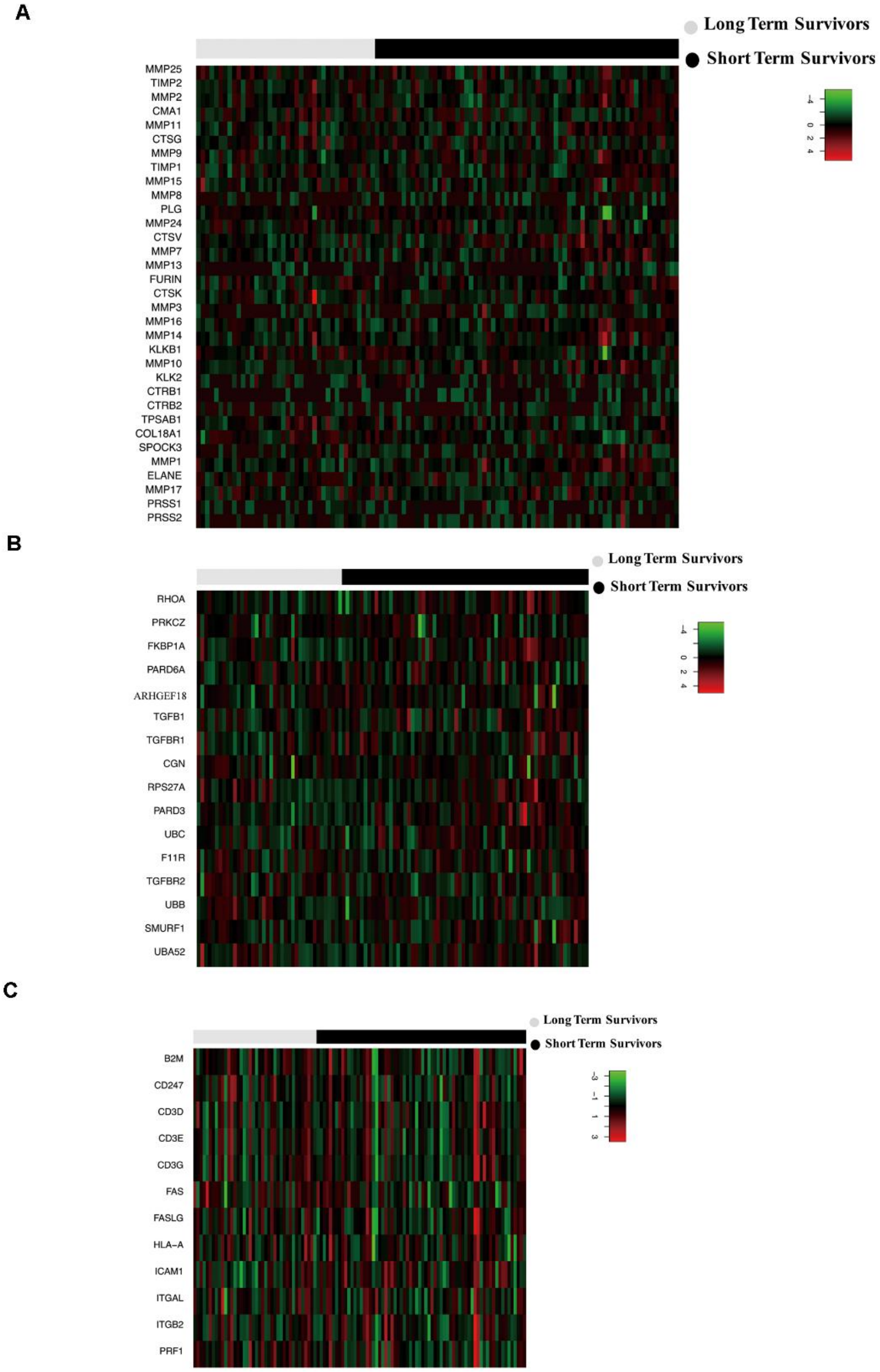

Supplementary Figure 1. Tumor sample expression signature heatmaps of three pathways related to cancer metastasis and immune responses between STSs and LTSs of HCC. (**A**) Reactome pathway: Activation of Matrix Metalloproteinases. (**B**) Reactome pathway: TGF-beta receptor signaling in epithelial to mesenchymal transition. (**C**) Biocarta pathway: CTL mediated immune response against target cells. Rows indicate the identity of the genes and columns indicate the identity of the patients. The survival status for each tumor is depicted directly above each column.

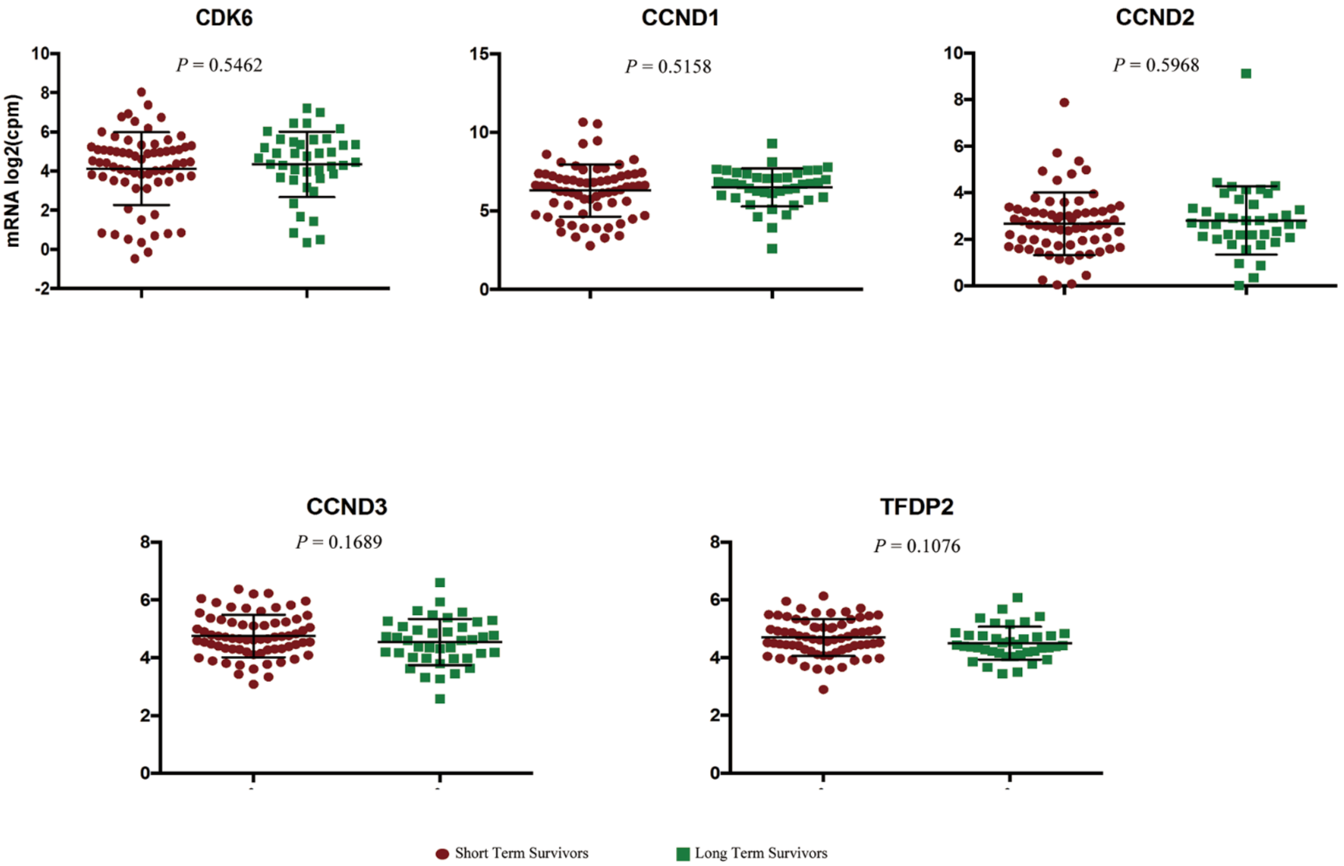

Supplementary Figure 2. Tumor sample transcriptomic profiling of five statistic non-significant positive regulators of G1/S cell cycle transition between STSs and LTSs of HCC. The data are represented as mean ± SD and compared by unpaired two-tailed Student's t-test. cpm, counts per million.

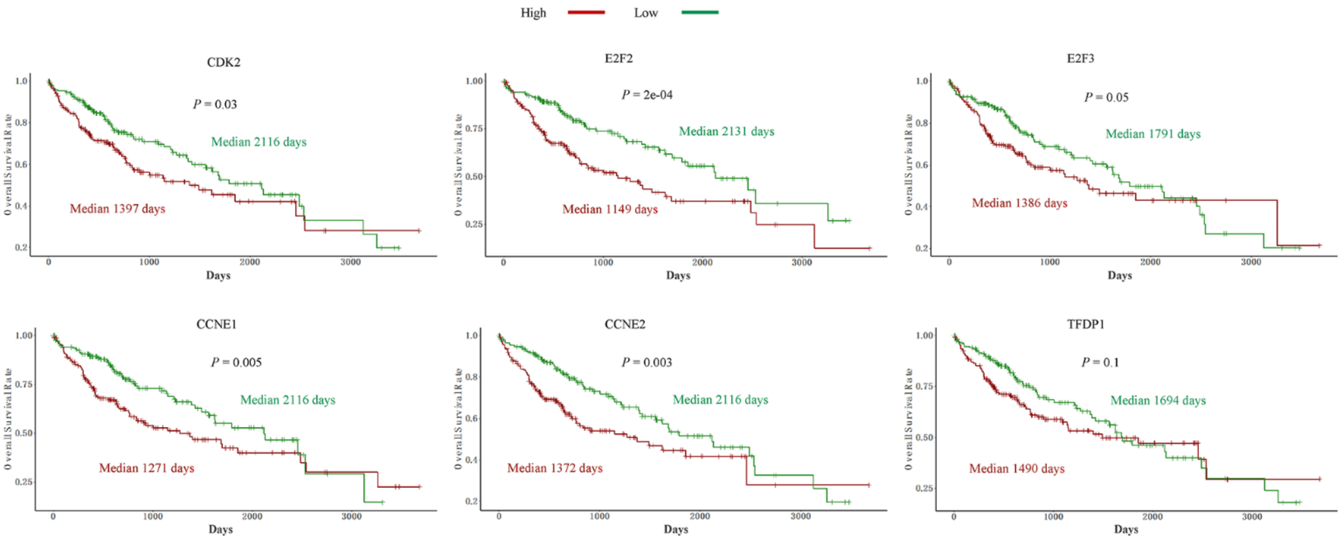

Supplementary Figure 3. Kaplan-Meier curves and log-rank tests for 342 HCC patients from LIHC project in TCGA database. The patients were stratified into the high expression group and the low expression group according to the median of normalized RNA-seq data.
